# Tropane and Pyrrolizidine Alkaloids in Edible Flowers and Flower-Derived Foods: A Food Safety Perspective

**DOI:** 10.3390/foods14213695

**Published:** 2025-10-29

**Authors:** Begoña Fernández-Pintor, Sonia Morante Zarcero, Isabel Sierra

**Affiliations:** 1 Departamento de Tecnología Química y Ambiental, Escuela Superior de Ciencias Experimentales y Tecnología (E.S.C.E.T.), Universidad Rey Juan Carlos, C/Tulipán s/n, Móstoles, 28933 Madrid, Spain; begona.fernandez@urjc.es; 2 Instituto de Tecnologías para la Sostenibilidad, Universidad Rey Juan Carlos, C/Tulipán s/n, Móstoles, 28933 Madrid, Spain

**Keywords:** edible flowers, tropane alkaloids, pyrrolizidine alkaloids, food safety, risks

## Abstract

The consumption of edible flowers has gained increasing global attention, driven by the demand for natural and functional foods. Edible flowers are consumed in various forms, including fresh, dried, or as ingredients in derived products such as infusions, dietary supplements, and honey. Their growing popularity is associated not only with their ability to enhance sensory properties, such as aroma, color, and flavor, but also with their potential health-promoting effects. Nevertheless, their consumption entails safety concerns related to possible contamination with pesticide residues, heavy metals, insects, microorganisms, and naturally occurring toxic compounds. Among these, tropane alkaloids (TAs) and pyrrolizidine alkaloids (PAs) represent major toxicological concerns. These alkaloids may be detected even in non-producing species due to cross-contamination in the field, horizontal transfer through soil, or pollination by bees that have previously visited TA- or PA-producing plants. This review addresses the risks associated with the consumption of edible flowers and flower-derived products, with particular emphasis on studies published since 2018. It provides an overview of the occurrence of TAs and PAs in fresh flowers, floral infusions, dietary supplements, and honey. Furthermore, it summarizes the analytical methodologies employed, including sample preparation and detection techniques, and compiles the reported concentrations of these alkaloids. The evidence presented highlights the need for continued investigation to establish reliable risk assessments and ensure consumer safety.

## 1. Introduction

**Figure 1:**
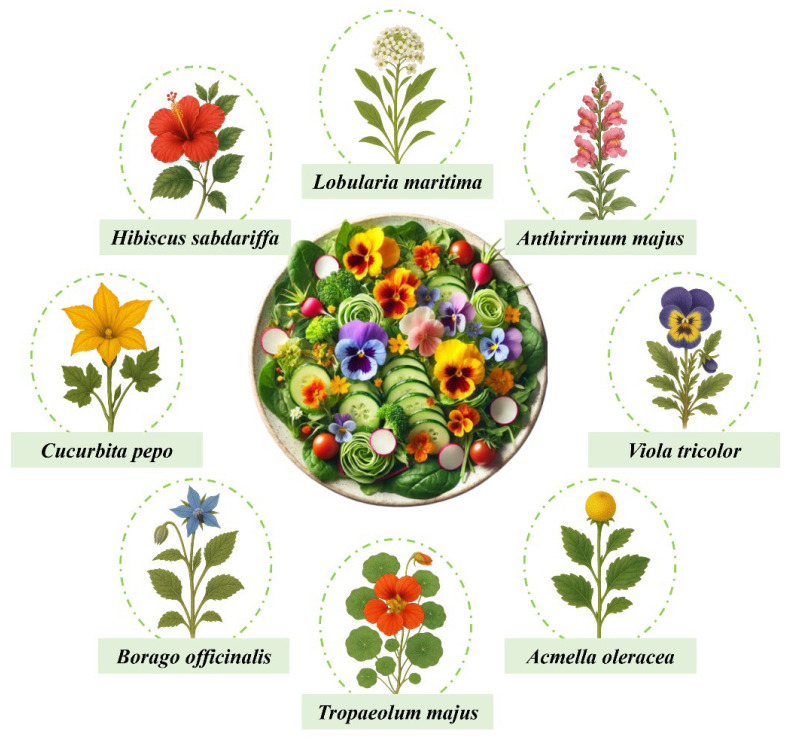
Some of the most commonly used edible flowers in gastronomy.

For millennia, various cultures have practiced the consumption of flowers, known as floriphagy, due to their culinary, medicinal, and aesthetic properties. More than 3000 years ago in China, chrysanthemum was used in medicinal infusions to improve eyesight, while jasmine was incorporated into aromatic teas. In India, hibiscus and rose were used in tonics and beverages, and the lotus flower held both spiritual and therapeutic value. In Rome and Greece, violet and rose were added to wines, honey, and as garnishes for certain dishes, while in Mexico, marigolds were used in various rituals. During the Medieval and Renaissance periods in Europe, liqueurs were prepared with lavender, rose, and calendula; in Japan, cherry blossoms were consumed as pickles and as part of traditional sweets [1]. In recent decades, edible flowers have gained remarkable prominence. Initially, renowned chefs incorporated them into their creations to surprise consumers with new gastronomic sensations, as they bring vivid colors, distinctive aromas, and unique flavors to dishes as illustrated in Figure 1 with some of the most commonly used edible flowers in gastronomy.

Later, flowers began to be commercialized so that consumers could prepare such dishes at home. Today, edible flowers are marketed in a variety of formats such as fresh, dehydrated, or crystallized, through specialized stores, supermarkets, and digital platforms. Their growing visibility on social media has significantly contributed to their consolidation as a gastronomic trend. The widespread circulation of images of flower-adorned dishes has had a notable impact on consumer perception, promoting interest not only in their organoleptic properties but also in their aesthetic value. This phenomenon has been identified as a factor influencing purchasing decisions, linking the visual appeal of food with the sensory and cultural experience of eating [2].

The consolidation of edible flowers as a gastronomic trend has been reinforced by studies analyzing consumer preferences. In a survey conducted in Belgium, 85% of consumers expressed interest in including flowers in their meals, and 83% had already consumed them. The most highly valued attributes were color and visual presentation, with warm tones such as yellow and orange being preferred over cooler tones like blue or purple. As for species, the most commonly used by chefs and consumers are wild pansy, borage, and nasturtium, although differences in perception were observed, consumers valued the flavor of wild pansy and borage more, whereas chefs preferred nasturtium for its aromatic intensity and visual appeal. Moreover, the sales format influences purchasing decisions, with small mixed-flower packages being the most attractive. These findings highlight how sensory and visual components drive demand, fostering market expansion and diversification of culinary applications [3,4,5,6]. The production of edible flowers faces significant limitations due to their high perishability, which conditions both handling and commercialization. Their shelf life, generally 7 to 10 days, requires careful harvesting during the coolest hours of the day, followed by immediate cooling to preserve sensory properties. Furthermore, they must typically be delivered within 3 to 4 days, obliging producers to make frequent deliveries in small quantities, particularly to restaurants. To extend shelf life, storage at 1–5 °C in perforated containers to prevent condensation is recommended, although even under these conditions issues such as wilting, discoloration, and oxidation may arise. Variability among species in terms of seasonality and postharvest resistance adds complexity to the process, hindering the standardization of storage and distribution protocols [7].

Another factor driving flower consumption, beyond their sensory qualities, is the growing interest in their potential health benefits, aligned with the trend toward more natural and organic diets. Numerous studies have focused on analyzing the bioactive compounds present in these flowers, highlighting their functional and therapeutic potential. Edible flowers contain a wide range of substances with antioxidant, antimicrobial, and even anticancer properties. Among these, phenolic compounds are particularly relevant, as they are present in high concentrations and strongly associated with health-promoting effects. They include flavonoids such as quercetin, rutin, kaempferol, luteolin and apigenin, and phenolic acids such as caffeic, ferulic, chlorogenic, vanillic, salicylic and p-coumaric acids, as well as tannins and other polyphenols. Depending on the species, total phenolic content can vary [8]. For example, in *Calendula officinalis*, quercetin, rutin and luteolin are major phenolics, with total contents of 25–40 mg gallic acid equivalents (GAE) per gram of dry weight (DW), together with carotenoids such as lutein, zeaxanthin and β-carotene, which enhance both nutritional and functional value [9]. Similarly, *Acmella oleracea* is rich in caffeic, ferulic, vanillic, isoferulic and chlorogenic acids, along with quercetin, rutin, apigenin and miquelianin; its phenolic content typically ranges between 15 and 35 mg GAE/g DW [10]. In *Viola tricolor*, concentrations are often slightly higher (20–40 mg GAE/g DW), with flavonoids (quercetin, kaempferol, violanthin), anthocyanins (delphinidin, cyanidin) and phenolic acids (salicylic, p-coumaric, ferulic) contributing to its strong antioxidant profile [11]. *Rosa* spp. also contains abundant phenolics (15–30 mg GAE/g DW), mainly gallic acid and quercetin derivatives, as well as anthocyanins, tannins and vitamin C (up to 200–250 mg/100 g fresh weight (FW)), reinforcing its role as a natural antioxidant source [12]. In *Tagetes erecta*, chlorogenic and gallic acids are present along with high levels of lutein (4–8 mg/g DW) and other carotenoids, with total phenolic content typically around 10–25 mg GAE/g DW [13]. The biological activities of these compounds explain the growing interest in edible flowers as functional ingredients. Their antioxidant action is closely linked to the presence of polyphenols and carotenoids, which contribute to cellular protection against oxidative stress. In addition, many species show anti-inflammatory, antimicrobial, wound-healing, antidiabetic, hepatoprotective, cardiovascular, neuroprotective and even chemopreventive properties, often through the modulation of enzymes such as amylase and lipase or the regulation of oxidative pathways. The pigments responsible for flower color, particularly carotenoids and anthocyanins, not only enhance their visual appeal but also act as markers of high bioactive content. Altogether, these characteristics position edible flowers as promising natural sources of health-promoting compounds and valuable ingredients for the development of functional foods and nutraceuticals [8,9,10,11,12,13].

In this context, interest in edible flowers is not limited to their consumption in fresh form. Increasing attention is being given to their derivatives, both in direct forms (such as infusions, extracts, essential oils, or dietary supplements containing edible flowers) and indirect forms, with honey being one of the most representative examples, as it is a product made up of bees from floral nectar and closely linked to the pollination process. These products expand the potential use of the bioactive compounds present in flowers, facilitating their incorporation into daily diets. However, despite their multiple benefits and wide range of applications, the consumption of edible flowers and their derivatives is not without risks. Factors such as the presence of naturally occurring toxic compounds, pesticide residues, microbiological contamination, or potential allergies must be carefully evaluated. Furthermore, the absence of specific regulation and standardized criteria for their production and commercialization may compromise consumer safety.

In this context, the present review aims to provide a comprehensive examination of the potential hazards associated with the consumption of edible flowers, thereby contributing to their safe and responsible integration into the food sector. Particular emphasis is placed on the occurrence of alkaloids in both flowers and flower-derived products, including floral infusions, food supplements containing different flowers, and honey. Special consideration is given to the presence of tropane alkaloids (TAs) and pyrrolizidine alkaloids (PAs), based on studies published between 2018 and the present. A critical evaluation of the analytical methodologies employed, as well as the reported levels of these alkaloids, is conducted to assess their potential implications for public health.

## 2. Contaminants and Hazards in Fresh Edible Flowers

The cultivation of flowers, particularly those intended for human consumption, may involve chemical, physical, and microbiological hazards. These risks can arise at different stages of the process, from cultivation and harvesting to storage and commercialization. Their identification and control are essential to ensure the safety of edible flowers and protect consumer health. In this context, official monitoring systems, such as the Rapid Alert System for Food and Feed (RASFF), have reported several incidents related to edible flowers [14]. Table 1 summarizes the alerts reported in recent years for food products, classified by contaminant type.

As can be seen in Table 1, recent notifications from the RASFF concerning dried flowers and flower-derived products intended for infusion [14]. These alerts were predominantly issued by Germany, Austria, France, Italy, and Switzerland, and involved several producing and exporting countries, including Egypt, Bulgaria, Albania, and Nigeria. Within the pesticide category, numerous non-compliances were identified due to the presence of unauthorized substances in products labeled as organic. Detected compounds included ethylene oxide, prometryn, chlorpyrifos, and thiophanate-methyl, all of which are prohibited in the European Union owing to their toxicological impact on human health and the environment. The associated risks were classified as serious or potentially serious. Consequently, corrective measures comprised immediate market withdrawal, notification of competent authorities, communication to recipients, and, in certain cases, destruction of contaminated batches. Regarding microbiological hazards, the presence of *Salmonella* spp. and *Salmonella* Typhimurium was confirmed in red clover, lavender, and linden flowers, primarily originating from Albania, France, and Bulgaria. Although some notifications were not deemed critical, all cases required product retention, application of physical decontamination treatments, and official reporting to mitigate the risk of foodborne illness. In the alkaloid category, a case involving excessive concentrations of pyrrolizidine alkaloids was documented in chamomile flowers sourced from Egypt. These naturally occurring compounds, present in certain plant species, exhibit hepatotoxic properties when ingested in high amounts. As a result, the implicated product was withdrawn from the market and consumer advisories were issued. For that reason, these notifications underscore the critical importance of stringent sanitary and phytosanitary controls in flower-based products intended for infusion, including those marketed as organic. The reported incidents highlight the necessity of reinforcing surveillance systems, traceability protocols, and analytical controls for pesticide residues and microbiological contaminants, thereby ensuring consumer safety and maintaining confidence in natural products.

These notifications indicate that pesticide contamination represents one of the most significant chemical hazards in edible flowers and derivative products. They are used to control pests and diseases during cultivation, but their indiscriminate application may leave harmful residues in flowers. These compounds are associated with genotoxic, neurotoxic, endocrine-disrupting, and reproductive effects. In the European Union, pesticide residues are regulated under Regulation (EC) No 396/2005, which establishes MRLs for herbs and edible flowers [15]. In the study by Bordoloi et al. (2024), 96 pesticides were analyzed in *Begonia palmata* using liquid chromatography coupled with mass spectrometry (LC-MS/MS), and all were found below the detection limit (LOD = 0.01 mg/kg), indicating no contamination [16]. In contrast, Pereira et al. (2021) analyzed ornamental flowers and detected 94 pesticides banned in the European Union, with concentrations up to 97 mg/kg in flowers and gloves. Severe effects on humans and wildlife were documented, and the lack of MRLs for ornamental flowers was highlighted, which enables the circulation of contaminated products. These findings emphasize the importance of using flowers specifically cultivated for human consumption, under sustainable and certified agricultural practices. While many producers employ organic agriculture, avoiding synthetic pesticides and fertilizers, it is crucial to verify product traceability and certification [17].

In addition to pesticide residues, other chemical hazards of concern in edible flowers include naturally occurring toxins, particularly mycotoxins and alkaloids. These compounds are secondary metabolites produced by fungi and plants, respectively, and may pose significant health risks. While information on mycotoxins in fresh or dried flowers is still limited, available data suggest that contamination could occur during cultivation, drying, or storage under inadequate conditions. Conversely, data on alkaloids are more abundant and will be discussed in detail in the following section. Both groups of compounds are relevant from a toxicological perspective, as they can cause hepatic, neurological, or carcinogenic effects depending on the type and concentration of toxin.

Although no recent RASFF alerts have been issued specifically for heavy metals, their presence remains a potential toxicological concern in edible flowers. Heavy metals can accumulate in plant tissues and may appear in edible flowers due to soil and water contamination, as well as through the use of fertilizers and soil amendments containing trace amounts of these elements. This accumulation occurs via root absorption and translocation to aerial parts of the plant, including flowers, leaves, and stems. Their presence in edible flowers constitutes a significant toxicological concern, as many heavy metals exhibit bioaccumulative properties, progressively accumulating in the human body and resisting metabolic elimination. Documented adverse effects include renal, hepatic, neurological, reproductive, and carcinogenic damage, depending on the metal, the dose, and the duration of exposure. For example, cadmium can interfere with kidney and bone function, lead affects the central nervous system, particularly in children, and arsenic is associated with various types of cancer [18,19,20]. In addition, metals such as chromium and nickel, although they may have biological functions at very low doses, become toxic when regulatory thresholds set by organizations such as World Health Organization (WHO) or European Food Safety Authority (EFSA) are exceeded. The presence of heavy metals in food is regulated in the European Union by Regulation (EU) 2023/915, which sets maximum levels for contaminants such as lead, cadmium, mercury, and arsenic [21]. Monitoring these elements in edible flowers is therefore essential, especially in products intended for direct consumption or medicinal use. Two recent studies evaluated the presence of heavy metals in edible flowers. Dos Santos et al. (2018) analyzed rose petals using inductively coupled plasma mass spectrometry (ICP-MS), detecting only trace amounts of toxic metals. Specifically, arsenic was detectable in a single sample at 0.02 mg/kg, while cadmium and chromium were below the quantification limit (LOQ). Cobalt levels ranged between 0.001 and 0.01 mg/kg, molybdenum between 0.02 and 0.2 mg/kg, and nickel between 0.04 and 0.2 mg/kg, values considerably lower than those typically found in other plant-derived foods such as tea or leafy vegetables. All concentrations were well below the Maximum Residue Levels (MRLs) established by international regulations, including those of ANVISA (Brazilian Health Regulatory Agency) and the European Union (e.g., Regulation (EU) 2023/915), indicating that the rose petals analyzed are safe for human consumption across multiple regulatory frameworks [22]. Conversely, Bordoloi et al. (2024) conducted a toxicological analysis of *Begonia palmata* leaves and petioles using ICP-MS, following Food Safety and Standards Authority of India (FSSAI) and WHO guidelines. Although the study focused on leaves, the results are extrapolatable to flowers, as they share the same growing environment and plant metabolism. Most heavy metals were within permitted limits, except for chromium (4.191 mg/kg) and nickel (2.942 mg/kg), which exceeded the established thresholds (1.0 and 1.5 mg/kg, respectively). Although the authors noted possible beneficial effects at controlled doses, these findings justify a deeper evaluation of their toxicity [16].

In addition to chemical hazards, microbiological contamination represents an important safety concern in edible flowers, particularly when cultivation or post-harvest handling occurs under inadequate hygienic conditions. These risks include contamination by pathogenic bacteria such as *Salmonella* spp., *Escherichia coli*, *Staphylococcus aureus*, as well as yeasts and molds, which may cause foodborne illnesses. Microbiological hazards in edible flowers are not yet subject to specific legislation. However, under Regulation (EC) No 852/2004, producers are required to apply Good Agricultural Practices (GAPs) and GHPs to minimize contamination, with particular emphasis on water quality, proper composting of manure, and safe handling [23]. This is particularly relevant given that edible flowers are commonly consumed raw and without further processing. The practical relevance of these risks has been confirmed by the RASFF, which recently reported two alerts concerning the presence of *Salmonella* spp. in edible flowers, specifically lavender and red clover flowers, as well as another alert related to *Salmonella* Typhimurium in linden flower infusions (Table 1). These findings underscore that microbiological hazards are not only theoretical but have already reached the consumer market. Accordingly, two recent studies have demonstrated the presence of these microorganisms in edible flowers. A study conducted in Poland analyzed five types of edible flowers: nasturtium (*Tropaeolum majus*), calendula (*Calendula officinalis*), daylily (*Hemerocallis*), daisy (*Bellis*), and carnation (*Dianthus caryophyllus*). *E. coli* was detected in nasturtium flowers grown in soils fertilized with manure, highlighting the risk associated with inadequately composted organic fertilizers. In addition, *Staphylococcus aureus* was present in all samples. Although hygienic harvesting slightly reduced microbial loads, the study concluded that pre-harvest factors such as soil type and irrigation water were more determinant than the harvesting method [24]. Wetzel et al. (2010) examined the microbial diversity of organic edible flowers (mainly *Viola tricolor*, *Pelargonium*, and *Tropaeolum*) and basil (*Ocimum basilicum*), cultivated under both organic and conventional systems. Potentially pathogenic bacteria such as *Salmonella enterica*, *Pseudomonas aeruginosa*, *Bacillus amyloliquefaciens*, and several Enterobacter species were identified. In particular, *Salmonella enterica* was detected in one organic sample, evidencing a relevant microbiological risk. Moreover, organic samples exhibited greater microbial diversity, possibly due to manure use and increased environmental exposure [25]. Both studies agree that, since edible flowers are typically consumed raw and uncooked, strict hygienic measures are critical during production, including the use of clean water, proper composting of manure, and safe harvesting practices. In addition to these preventive measures at the primary production stage, post-harvest sanitation plays a crucial role in minimizing microbial contamination. Current food safety guidelines in the European Union and recommendations from the WHO support washing with clean water and, when disinfection is required, the use of chlorine-based solutions or other approved sanitizers. When chlorine is used, its concentration and contact time must be carefully controlled to avoid excessive residual levels that could compromise product quality and consumer safety. Typically, concentrations of 50–200 mg/L of free chlorine are considered effective for fresh produce, provided that the product is subsequently rinsed with potable water to remove any residues. Failure to adequately control chlorine levels may lead to sensory alterations or the formation of potentially harmful disinfection by-products. Washing and disinfecting flowers before consumption, even when sourced from organic production, is therefore recommended to reduce the risk of foodborne disease, provided that the process is properly managed [23].

Finally, physical hazards include the presence of foreign materials such as insects, hard plant fragments, soil, or stones. Although these contaminants do not pose chemical or microbiological toxicity, they can cause physical injury or lead to product rejection by consumers. This issue is particularly relevant in the context of organic or low-input agricultural practices, which aim to promote sustainability and reduce chemical risks through minimal or no use of synthetic pesticides. However, such production systems may increase the likelihood of detecting insects or other natural elements in the harvested flowers. Although there are no specific regulations for physical contaminants in edible flowers, general food hygiene legislation, particularly Regulation (EC) No 852/2004 on the hygiene of foodstuffs, requires producers to minimize the presence of foreign matter through Good Handling Practices (GHPs). For this reason, some commercially available packages of fresh edible flowers include labeling statements indicating that small insects may occasionally be present, due to the absence of synthetic pesticides during cultivation. To address this, producers often recommend immersing the product in cold water with the addition of approximately 2% white wine vinegar for about 15 min prior to consumption. It is important to note that this practice is intended merely to aid in the removal of insects or other physical debris and does not constitute a recognized disinfection procedure according to food safety standards [23].

In summary, the presence of chemical, microbiological, and physical hazards in edible flowers underscores the importance of implementing strict monitoring and control measures throughout the production chain. Current European legislation provides partial regulation, covering pesticide residues, heavy metals, and general food hygiene, but specific standards for edible flowers are still lacking. Since these products are often consumed raw and without further processing, even minimal contamination may pose significant health risks. Ensuring safe cultivation practices, proper handling, and effective quality control is therefore essential not only to protect consumer health but also to strengthen consumer confidence and support the sustainable development of the edible flower market. The repeated occurrence of RASFF alerts involving both pesticides and microbiological hazards in edible flowers confirms the urgent need for harmonized standards and more rigorous monitoring across the European Union.

## 3. Natural Toxins in Flowers and Flower-Based Products: Occurrence and Risks of Pyrrolizidine and Tropane Alkaloids

Edible flowers may contain a wide range of toxic secondary metabolites, which are naturally produced as part of the plant’s metabolism. While some of these compounds contribute to beneficial health effects, others can pose toxicological risks under certain conditions. In addition, the visual similarity between edible and poisonous flowers introduces an extra hazard related to misidentification.

**Figure 2:**
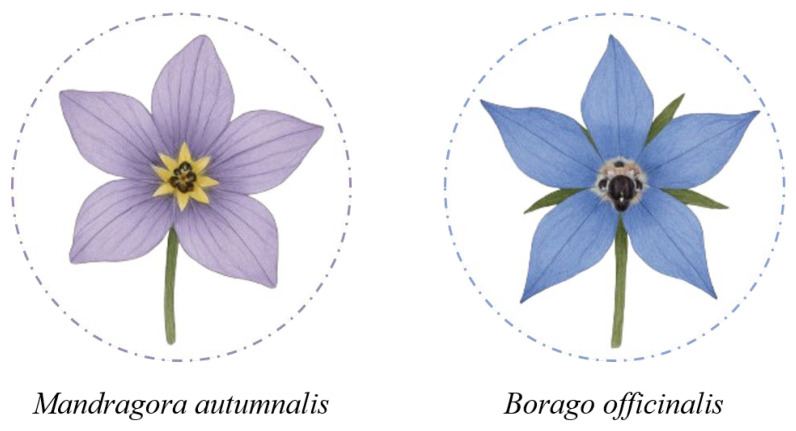
Morphological similarity between the floral species *Mandragora autumnalis* and *Borago officinalis*.

As with mushroom foraging, where the risk of confusing edible species with toxic ones is well recognized, the collection of wildflowers for human consumption can also pose serious hazards if conducted without proper botanical knowledge. Although many flowers are prized for their culinary and nutritional properties, identifying them in the wild can be challenging, particularly in spring, when young shoots and leaves often exhibit similar morphologies across different species. The study by Colombo et al. (2010) documents numerous cases of poisoning due to confusion between edible flowers and toxic plants, such as wild garlic (*Allium ursinum*), whose leaves and flowers are prized in cooking, but which has been mistaken for lily of the valley (*Convallaria majalis*), containing cardioactive glycosides like convallarin, and autumn crocus (*Colchicum autumnale*), rich in colchicine, a highly toxic and potentially lethal alkaloid. Similarly, borage (*Borago officinalis*), used in soups and ravioli, has been erroneously collected as autumn mandrake (*Mandragora autumnalis*), a plant containing TAs such as atropine and scopolamine, which are responsible for severe anticholinergic symptoms (Figure 2). Poisonings have also been reported from the use of elderflowers (*Sambucus nigra*) mistaken for species within the same genus, such as *S. racemosa* and *S. ebulus*, which contain sambunigrin, a cyanogenic glycoside. These confusions, driven by superficial similarities in color, shape, or flowering time, highlight the need for precise and responsible identification, particularly when it comes to wildflowers intended for human consumption. For this reason, it is recommended to acquire edible flowers only from specialized establishments, where proper identification, traceability, and food safety are guaranteed [26].

Among the various classes of potentially hazardous phytochemicals, PAs and TAs have received particular attention in recent years due to their occurrence in edible plants and derived products. These nitrogen-containing compounds are primarily synthesized by plants as defense molecules, and their presence in food has prompted increased interest in monitoring and regulating their levels, with maximum limits recently established for several food categories. In humans, PAs and TAs can cause a wide range of toxic effects, often at very low doses.

Alkaloids can be classified into several structural groups, each exhibiting distinct toxicological profiles, with PAs representing one of the most relevant groups due to their well-documented toxicity. These compounds are found in species such as *Borago*, *Senecio*, and *Crotalaria*, and are known to be hepatotoxic, genotoxic, and potentially carcinogenic. Their *N*-oxide forms (PANOs) also contribute to toxicity, as they can be reduced to the parent alkaloids in the gastrointestinal tract, thereby increasing overall exposure. Chronic low-level exposure to PAs and PANOs can lead to veno-occlusive disease and liver failure. Many countries regulate the content of PAs and PANOs in foodstuffs; for example, the European Union has set maximum limits for total PAs in teas and herbal infusions, and EFSA provides intake guidance to minimize chronic exposure [27,28,29]. Under Regulation (EU) 2023/915, maximum limits have been established for 21 PAs and PANOs in several food categories, including borage fresh leaves, dried herbs, certain spices, teas and herbal infusions, cumin, as well as food supplements containing plant preparations (including extracts) and pollen-based products. The limits are 200 µg/kg for dried materials intended for herbal infusions, although in some cases, such as chamomile, higher thresholds of 400 µg/kg apply; food supplements are also subject to 400 µg/kg. Importantly, edible flowers are not explicitly included, although flower-derived supplements and flower infusions could potentially fall under the regulated categories [17]. While edible flowers, in general, are not explicitly regulated and there have been no widespread alerts for PAs, in 2025 a case was reported for chamomile flowers containing PAs, highlighting that even flowers considered safe may pose a risk (alert reference: 2025.1005) [14]. For other edible flowers, no alerts have been reported, likely due to the lack of specific regulation (Table 1).

TAs, such as atropine and scopolamine, occur naturally in several species of the Solanaceae family, including *Datura stramonium*, *Atropa belladonna*, and *Hyoscyamus niger*. These compounds primarily affect the autonomic nervous system, producing anticholinergic effects. Toxic manifestations can include dry mouth, blurred vision, tachycardia, hallucinations, seizures, and, in severe cases, death. TAs are rapidly absorbed from the gastrointestinal tract, and their toxicity depends on factors such as dose, age, and overall health status [30,31]. Regulation (EU) 2023/915 specifically addresses atropine and scopolamine, the two most prevalent and toxicological relevant TAs in foods. The strictest limits are applied to cereal-based infant foods due to the high vulnerability of infants. Other regulated matrices include millet, sorghum, maize, and buckwheat grains and their derived products, as well as dried herbal infusions and liquid herbal infusions. Maximum levels are set at 25 µg/kg for dried herbal materials and 0.2 µg/L for liquid infusions. These regulatory measures aim to minimize dietary exposure to TAs and protect sensitive consumers, particularly infants and young children, from potential acute and chronic toxic effects.

While flowers themselves are not explicit mentioned in legislation, flower-based infusions could be considered within the broader category of herbal infusions. Alerts have been reported for PAs and TAs in herbal infusions; however, these notifications do not specify that the products were derived from flowers. Similarly, food supplements containing plant extracts (including potential flower extracts) have been subject to alerts for PAs and TAs, yet there is no direct evidence implicating flowers as the source [17].

The intake of PAs and TAs through the consumption of fresh or dried flowers can result, on the one hand, from the natural presence of these compounds in the edible species themselves, as is the case with *Borago officinalis* or *Tussilago farfara*, which are commonly consumed in significant amounts. Even when the flowers belong to species not known to produce alkaloids, toxic compounds can still be transferred if they grow in close proximity to poisonous plants or share soil and root exudates, a process particularly relevant for alkaloids, which can accumulate at low levels in neighboring edible plants [32,33,34]. In derived products such as teas, herbal infusions, and food supplements, contamination may also result from residual material of misidentified flowers, as well as cross-contamination during processing, storage, or transport. Cross-contamination in this context refers to the unintentional transfer of alkaloid-producing plant material into otherwise safe products. This can occur already at the agricultural stage, when edible flowers are cultivated in fields where toxic species also grow. The use of automatic harvesting machinery, such as combine harvesters, increases this risk, as edible and toxic plants may be collected together and enter the same processing chain [35,36,37]. These contamination pathways, combined with the toxicological properties of the alkaloids, underscore the importance of developing sensitive and selective analytical methods. Such methods are crucial because alkaloids are often present at very low concentrations and edible flowers are chemically complex matrices rich in other phytochemicals. Rigorous phytochemical analysis, together with strict compliance with existing regulatory limits, is essential to ensure the safe consumption of edible flowers and their derived products.

Table 2 summarizes the analytical procedures applied and the concentration levels reported for edible flowers and flower-derived products such as flower-based infusions and food supplements. The presence of these toxic secondary metabolites in honey and honey-based products will be discussed in subsequent sections. This compilation provides an overview of current knowledge and illustrates the variety of methodologies used for their detection and quantification.

### 3.1. Occurrence and Analytical Determination of Pyrrolizidine and Tropane Alkaloids in Edible Flowers

Despite the recognized toxicological relevance of PAs and TAs, targeted studies focusing on their occurrence in fresh flowers remain scarce. This research gap is partly attributable to the absence of specific regulatory frameworks governing edible flowers, which has limited the amount of systematic toxicological studies conducted. Consequently, most available investigations have centered on broad phytochemical screenings rather than on specific alkaloid identification and quantification.

The majority of published studies employ classical qualitative phytochemical tests, including Mayer’s, Dragendorff’s, Wagner’s, and Hager’s reagents, or thin-layer chromatography (TLC), which enable only the detection of total alkaloids without providing detailed information on individual compounds or precise concentration levels [70,71,72,73,74,75]. Some studies also apply simple colorimetric or precipitation-based assays as preliminary screens for alkaloid presence. Such approaches have reported alkaloids in species such as *Acmella oleracea* and *Cucurbita maxima*, although results are often inconsistent. These discrepancies can be attributed to differences in extraction solvents, sample treatment (e.g., fresh versus dried material), extraction time, and analytical sensitivity. Moreover, most of these studies focus on the bioactive potential of alkaloids, such as antioxidant, antimicrobial, and anti-inflammatory properties, rather than their toxicological implications. This lack of toxicological focus limits the usefulness of the data for risk assessment and regulatory considerations, highlighting the need for more targeted and quantitative analyses of specific alkaloids in edible flowers.

In contrast, only a limited number of studies have applied validated analytical methodologies to detect and quantify individual alkaloids in edible flowers (Table 2) [38,39,40,41]. Most of these works used solid–liquid extraction (SLE) with acidified solvents, such as methanol–water mixtures or diluted sulfuric or formic acid, followed by chromatographic analysis using liquid chromatography coupled to mass spectrometry (LC-MS/MS) or ultra-high-performance liquid chromatography–quadrupole time-of-flight mass spectrometry (UHPLC-Q-TOF/MS) [38,40,41]. In one case, the extract was further purified by solid-phase extraction (SPE) using a C18 cartridge prior to instrumental determination [40]. A different study employed high-pressure extraction (HPE) with acetic acid instead of conventional SLE [39]. As summarized in Table 2, all studies utilized reversed-phase C18 columns, generally maintained between 25 and 40 °C, under positive electrospray ionization (ESI+) and MRM (multiple reaction monitoring) mode. These methodological consistencies indicate that C18-based chromatographic systems provide adequate selectivity and sensitivity for the determination of PAs in complex floral matrices. Nevertheless, this approach may present limitations in the resolution of some isomeric PAs and therefore may not be universally applicable to all legislated compounds.

Among the analyzed species, *Borago officinalis* (borage) consistently exhibited the highest PA content. Depending on the part of the plant analyzed, between 9 and 18 individual PAs were identified, with total concentrations ranging from 77 to 88,602 µg/kg DW [38,40,41]. In these studies, recoveries typically ranged between 85 and 112%, confirming good method performance (Table 2). By contrast, *Chrysanthemum morifolium* flowers showed the presence of only one PA [39]. The differences observed across these works can be attributed to both species-dependent alkaloid profiles and variations in extraction and purification strategies, particularly regarding solvent composition, extraction strength, and cleanup step. From a toxicological perspective, the concentrations reported for borage flowers greatly exceed existing regulatory limits for borage leaves (750 µg/kg FW) [41,42]. Considering the typical moisture content of floral tissues (80–90%), the dry-weight concentrations reported in the literature (up to 88,602 µg/kg DW) correspond to approximately 8860–17,720 µg/kg FW, still well above the permitted threshold. Even moderate consumption of these flowers could therefore surpass the tolerable daily intake (TDI) for PAs established by EFSA. Importantly, the detection of PAs in *Borago officinalis* flowers is consistent with the species’ phytochemical profile, as these compounds are endogenously produced and naturally distributed throughout aerial organs, including floral tissues. Thus, their presence does not necessarily indicate contamination but reflects intrinsic biosynthesis that must be considered in risk assessment.

Overall, the available evidence demonstrates that toxic alkaloids can occur in both expected and unexpected floral matrices. However, the limited number of quantitative studies and the lack of methodological harmonization complicate direct comparisons and hinder robust exposure assessments. Future research should aim to standardize extraction and chromatographic conditions, as well as expand the analytical scope beyond traditionally studied species, to generate more reliable and comparable datasets for regulatory and toxicological evaluation.

### 3.2. Occurrence and Analytical Determination of Pyrrolizidine and Tropane Alkaloids in Edible Flower-Based Infusions

A comprehensive review of flower-derived products, including floral infusions and herbal teas containing flowers, reveals an extensive body of research addressing the occurrence of PAs and, to a lesser extent, TAs [42,43,44,45,46,47,48,49,50,51,52,53,54,55,56,57,58,59,60,61,62,63,64]. Studies have investigated both solid dry materials and liquid infusions, the latter being particularly relevant for estimating actual consumer exposure. Among edible flowers, *Matricaria recutita* (chamomile) is the most extensively studied species, followed by lavender (*Lavandula angustifolia*), hibiscus (*Hibiscus sabdariffa*), mallow (*Malva sylvestris*), calendula, rose hip, and linden (*Tilia* spp.).

As summarized in Table 2, PAs are frequently detected in floral infusions, particularly in chamomile-based preparations, where concentrations in dry products range between 4 and 700 µg/kg, with some studies reporting exceptionally high values exceeding 1000 µg/kg [42,47,54]. Upon infusion, transfer rates from dry material to liquid vary widely from 13% up to 87% depending on factors such as compound polarity, particle size, infusion pH, water temperature, and steeping time [42,45,47,60,64]. Hot water extraction consistently results in higher PA release than cold maceration, confirming that thermal extraction efficiency is a critical determinant of exposure.

Predominant PAs include senecionine, seneciphylline, europine, intermedine, and heliotrine, with *N*-oxide derivatives frequently representing the majority fraction [55,56,57]. In some single-flower infusions, concentrations in the final beverage exceed the European regulatory threshold of 5 µg/L for liquid infusions, revealing a tangible risk of consumer exposure. For instance, commercial chamomile teas have shown mean concentrations of 3–4 µg/L, with maxima up to 18 µg/L, equivalent to approximately 1400 µg/kg in dry tea [55]. TAs such as atropine and scopolamine are reported less frequently but can appear due to co-harvesting contamination from alkaloid-producing species (*Datura*, *Hyoscyamus*, *Atropa*) [43,51]. Although typical TA concentrations remain below regulatory limits (0.2 ng/mL), occasional exceedances have been reported, particularly under decoction conditions, which promote higher transfer rates [58,61]. Interestingly, flowers not naturally synthesizing these compounds, such as mallow or hibiscus, have occasionally tested positive, indicating environmental cross-contamination during cultivation or drying [57,59]. Both PAs and TAs exhibit substantial thermal stability during infusion and drying [60,61], reinforcing the necessity of evaluating the final beverage, rather than only the raw material, when assessing toxicological risk.

Analytical methodologies for the determination of PAs and TAs have evolved significantly over the past decade, reflecting both technological advancement and the complexity of floral matrices. Early studies primarily relied on SLE using acidified solvents such as methanol–water mixtures or diluted sulfuric/formic acid, followed by SPE cleanup on C18 or mixed-mode cation exchange (MCX) cartridges [44,45,46,47,50,52,53]. These procedures yielded recoveries between 52% and 152%, with LODs typically in µg/kg range. However, the efficiency of these classical methods depends heavily on matrix characteristics, floral materials being rich in essential oils, pigments, waxes, and polysaccharides that may interfere with extraction and ionization.

To address these challenges, QuEChERS (Quick, Easy, Cheap, Effective, Rugged, and Safe) method has been introduced and gained prominence, offering simplified, high-throughput extraction with effective cleanup using sorbents such as PSA, C18, ENVI-Carb, MgSO_4_, and GCB [42,48,51]. These approaches achieved recoveries around 85–110% and LODs as low as 0.03 µg/kg, confirming their suitability for complex herbal matrices. The integration of microextraction systems (µSPEed) and dSPE further improved selectivity while reducing solvent consumption, demonstrating good reproducibility (recoveries 79–97%) and applicability for both PAs and TAs [57,58,60,63]. Detection has also transitioned from TQ-MS/MS to high-resolution mass spectrometry (HRMS) platforms, such as Orbitrap or Q-TOF systems [48,49,62]. HRMS allows accurate mass determination, identification of *N*-oxide derivatives, and detection of low-abundance or unknown alkaloids, substantially improving analytical confidence. For instance, the HRMS-based method developed by Rizzo et al. (2023), employing salting-out assisted liquid–liquid extraction (SALLE) with MgSO_4_·7H_2_O (1 M) and Na_2_SO_4_ (1.5 M), and pH adjusted to 9.6 using NaOH (5 M), followed by liquid–liquid extraction with acetonitrile, enabled the detection of up to 118 distinct PAs in various edible flower infusions, far exceeding the compound coverage of traditional LC–MS/MS approaches [62].

The methodological comparison summarized in Table 2 illustrates a clear trend toward miniaturization, automation, and multi-analyte detection. Despite this progress, interlaboratory comparability remains limited due to differences in solvent composition, pH adjustment, cleanup sorbents, and chromatographic columns. Most studies employ C18 reversed-phase columns at 25–50 °C under positive electrospray ionization (ESI+) in MRM mode, ensuring high sensitivity and selectivity. Nonetheless, differences in ion suppression, matrix effect compensation, and calibration strategies still pose analytical challenges. When comparing methodologies, it is evident that sample preparation is the main determinant of quantitative variability. For example, the SLE–SPE-SCX (strong cation exchange) method applied to chamomile [44] provided high sensitivity but showed broader recovery variation (52–152%), suggesting partial matrix interference. In contrast, µSPEed (micro–solid phase extraction), which employs packed cartridges containing between 1 and 4 mg of sorbent material and operates with only a few microliters of sample and solvent, provides a rapid and highly efficient cleanup step. QuEChERS and µSPEed strategies combined with ultra-high-performance liquid chromatography–ion trap mass spectrometry (UHPLC–IT–MS/MS) [42,57,60,63] achieved more consistent recoveries (80–100%) and cleaner chromatograms, indicating superior matrix management. Similarly, HPLC–Q–Orbitrap HRMS platforms [48,62] delivered the most comprehensive alkaloid profiling, including minor or trace-level compounds previously undetected. In liquid infusion studies, the combination of boiling water extraction followed by µSPEed-C18 purification and UHPLC-IT-MS/MS detection [57,60,63] proved particularly efficient, with detection limits as low as 0.03 µg/L and recovery rates above 90%, supporting its use for accurate exposure assessment. These findings collectively underline the importance of selecting extraction and cleanup protocols tailored to the matrix and analytical objective.

Overall, the reviewed evidence demonstrates that although most commercial floral infusions comply with regulatory safety thresholds, substantial variability exists in contaminant levels and transfer efficiencies across species and preparation methods. Chamomile and lavender appear especially susceptible to PA contamination, whereas mallow, hibiscus, and calendula occasionally present detectable, though generally lower, levels. The data confirm that assessing the final infusion, rather than the raw dry material, provides a more realistic estimate of consumer exposure. From an analytical perspective, future progress requires method standardization and interlaboratory validation, particularly regarding extraction solvents, cleanup sorbents, and calibration strategies. The increasing use of HRMS-based non-targeted screening offers an opportunity to detect novel or emerging alkaloids, supporting both risk assessment and regulatory monitoring.

Finally, considering the persistence and thermal stability of these alkaloids, continuous surveillance and harmonized quality control protocols are essential, especially for products marketed as organic or consumed by vulnerable populations (children, pregnant women, individuals with liver impairment). A comprehensive risk assessment framework should integrate agronomic practices, postharvest contamination control, and advanced analytical chemistry, ensuring the safety and authenticity of flower-derived infusions within an increasingly globalized market.

### 3.3. Occurrence and Analytical Determination of Pyrrolizidine and Tropane Alkaloids in Edible Flower-Based Food Supplements and Extracts

The presence of PAs and TAs in herbal food supplements containing edible flowers has become an issue of increasing toxicological relevance. Compared to herbal teas or infusions, concentrated extracts and supplements represent a potential source of higher exposure, due to both accumulation effects during processing and the frequent inclusion of complex flower blends with variable botanical origins.

As summarized in Table 2, contamination levels in these products span a remarkably broad range from a few micrograms per kilogram up to over 200,000 µg/kg in extreme cases such as *Gynura japonica* extracts [66]. This wide variability reflects differences in species composition, geographical origin, harvesting practices, and post-harvest handling, as well as the potential for cross-contamination with alkaloid-producing weeds (e.g., *Senecio*, *Heliotropium*, *Borago*, *Datura*). Floral ingredients most frequently associated with PA occurrence include *Tussilago* spp., *Hypericum perforatum*, *Gynura japonica*, and dried *Matricaria recutita*, consistent with reports on herbal teas. The PA profiles are typically dominated by lycopsamine-type compounds and their *N*-oxides, with heliotrine, europine, echinatine, and senecionine derivatives also commonly reported, especially in supplements containing Boraginaceae and Asteraceae families [55,62,65,66,67,68]. In contrast, the detection of TAs such as atropine and scopolamine, even in the absence of known source plants, indicates that accidental contamination during harvesting, drying, or processing remains an underrecognized pathway for TA occurrence [43,68,69].

The analytical determination of PAs and TAs in these complex matrices presents notable challenges due to the heterogeneous composition, high extract concentration, and strong matrix effects typical of food supplements. Nevertheless, as shown in Table 2, recent studies have converged toward highly sensitive LC–MS-based platforms, often preceded by optimized extraction and cleanup steps tailored for floral matrices. Most works employed SLE with acidified solvents (e.g., H_2_SO_4_ 0.05 M, formic acid 0.2–1%) followed, in some cases, by liquid–liquid extraction (LLE) or SPE using C18, SCX, or MCX sorbents [55,65,66,67]. SCX and MCX sorbents are used in combination with specific pH conditions because they retain positively charged (protonated) alkaloids, allowing selective cleanup. At the appropriate pH, these compounds are ionized, which enhances their binding to the negatively charged functional groups on the sorbent, thereby reducing matrix interferences. One study uniquely implemented a SALLE step after SLE, which improved analyte recovery and reduced co-elution of pigments and lipophilic compounds [62]. Alternative sample preparation strategies such as QuEChERS (without cleanup) [68] and µSPEed microextraction using synthesized SM–C18 cartridges, packed with spherical mesostructured silica functionalized with C18 ligands synthesized in the laboratory, have shown high efficiency, offering recoveries between 85 and 110% for PAs and 90–94% for TAs. The µSPEed–UHPLC–IT–MS/MS method, in particular, demonstrated detection limits as low as 0.2 ng/g, with minimal solvent use and fast analysis times, representing a promising approach for high-throughput monitoring. Detection across all studies relied on electrospray ionization in positive mode (ESI+), under MRM or HRMS conditions, ensuring high selectivity and sensitivity. Columns were predominantly reversed-phase C18, operated at 25–50 °C. The use of UHPLC–Orbitrap–MS/MS for full-scan HRMS detection [62] allowed the simultaneous identification of up to 118 alkaloids, providing comprehensive chemical fingerprinting and improving confidence in compound annotation compared with conventional TQ-MS/MS.

Overall, the methodological trends summarized in Table 2 highlight a clear transition from traditional SLE–SPE workflows toward modern, miniaturized, and sustainable extraction techniques, coupled with advanced HRMS. These developments not only enhance analytical performance but also reduce environmental impact, sample requirements, and analytical time, key advantages for routine regulatory monitoring.

The contamination patterns reported in Table 2 indicate that concentrated liquid formulations, such as liqueurs, elixirs, and herbal juices, tend to accumulate higher PA levels (up to 3000 µg/kg) compared to powdered tablets or solid extracts [65]. However, even dry materials such as *Chrysanthemum morifolium* supplements or multi-flower blends frequently contain detectable PAs, in some cases exceeding 1000 µg/kg [67,68]. Processing factors, including drying, extraction solvents, and concentration steps, have a marked effect on alkaloid content. For instance, acidic alcoholic extractions enhance the solubilization of tertiary PAs and their *N*-oxides, while aqueous preparations preferentially extract polar PANOs. Consequently, liquid herbal extracts often exhibit higher apparent contamination levels per unit mass than solid matrices, despite similar raw ingredient profiles. Matrix complexity also influences analytical recovery: lipid-rich or resinous formulations can suppress ionization and reduce apparent concentrations, while dry powder supplements may retain strongly bound alkaloids in the plant matrix. Despite these differences, the overall PA:TA ratio remains highly skewed toward PAs, suggesting that PA contamination is the predominant chemical hazard in flower-based supplements.

Comparing analytical performance across the studies listed in Table 2 underscores that SLE followed by appropriate cleanup (SPE, SALLE, or µSPEed) provides the most consistent balance between recovery, selectivity, and throughput. While classical SLE–SPE–UHPLC–TQ-MS/MS methods remain reliable for routine quantification [55,65,67], recent HRMS-based workflows [62,69] allow for untargeted screening of minor or emerging alkaloids, an essential step for comprehensive risk assessment. The reported recovery rates (67–151%) and detection limits (0.03–30 µg/kg) demonstrate the high analytical sensitivity achieved by these methods. Importantly, the integration of miniaturized extraction approaches such as µSPEed also addresses environmental sustainability by reducing solvent consumption and waste generation, an increasingly relevant factor for large-scale surveillance programs. Nevertheless, differences in solvent systems, calibration procedures, and ionization efficiencies still limit cross-study comparability, emphasizing the need for standardized analytical protocols and certified reference materials for PAs and TAs in concentrated herbal matrices.

From a toxicological standpoint, the data summarized in Table 2 confirm that floral-based supplements can represent a meaningful exposure pathway to both PAs and, occasionally, TAs. Although most commercial products remain within regulatory thresholds, sporadic high concentrations, particularly in *Gynura japonica* and other Asteraceae-derived extracts, may exceed the European limit of 1 µg/kg body weight/day for total PA intake [66]. Moreover, the co-occurrence of PAs and TAs, as documented in several studies [68,69], raises concern for potential additive or synergistic toxic effects, which are rarely addressed in current risk assessments. Given the vulnerability of certain consumer groups, including children, pregnant women, and individuals consuming multiple herbal preparations simultaneously, continuous monitoring and risk characterization remain essential.

In summary, the occurrence data and methodological developments presented in Table 2 collectively demonstrate that flower-based food supplements and extracts can contain quantifiable levels of both PAs and TAs, with PA contamination being more widespread and variable. Despite methodological advances that enable detection at trace levels, heterogeneity in matrix composition, plant sourcing, and extraction conditions continues to drive substantial variability in reported concentrations. Looking ahead, research efforts should concentrate on establishing harmonized analytical standards and validated protocols specifically designed for supplement matrices, in order to improve comparability across studies and ensure data reliability. The incorporation of non-targeted HRMS approaches will further enhance the ability to profile both known and emerging alkaloids, supporting a more comprehensive toxicological evaluation. In parallel, attention should be given to understanding the potential transformations of these compounds during processing and storage, as such changes may alter their toxicokinetic properties and risk potential. Equally important is the development of eco-efficient analytical workflows that minimize solvent use and waste generation, aligning analytical monitoring with current sustainability goals. Ultimately, ensuring consumer protection in this sector requires an integrated strategy that combines robust analytical chemistry, toxicological risk assessment, and effective regulatory oversight. Strengthening these pillars will enhance the reliability of exposure estimates, support compliance with safety limits, and maintain public confidence in flower-derived food supplements.

### 3.4. Contamination of Honey, Pollen and Other Bee-Based Products Through Pollinating Insects: Occurrence and Analytical Determination of Pyrrolizidine and Tropane Alkaloids in These Products

Pollinating insects, such as bees, play a crucial role in the reproduction of flowering plants. However, they can also act as vectors for the transfer of toxic compounds, including PAs and TAs, from flowers to honey, pollen, and other bee-based products such as bee bread, royal jelly, and propolis. This pathway represents an additional route of human exposure to plant toxins, which may pose risks for food safety. In the context of bee-derived products, contamination through toxic compounds remains a concern. Under EU Regulation 2023/915, TAs are not specifically regulated in honey, pollen, or other bee-based products. For PAs, the regulation only sets limits for food supplements based on pollen, with a maximum of 500 µg/kg, leaving honey and pollen largely unregulated [17]. Despite this regulatory gap, alert data indicate that contamination issues do occur in these products, with recorded alerts including hydroxymethylfurfural (3), grayanotoxins (4), mycotoxins (1), unauthorized substances (12), antibiotics (10), and physical contamination or poor hygiene (4). Notably, there are no recorded alerts for alkaloids in these products, which may reflect the lack of specific legislation and systematic monitoring rather than absence of risk [12].

Several studies have addressed the presence, stability, and detection of PAs in bee products, providing key insights for food risk assessment (Table 3). While the literature on PAs is extensive and growing, research on TAs in apicultural matrices remains limited. This disparity is likely due to the lack of specific legislation or regulatory thresholds for TAs in honey and related products, which may have hindered systematic monitoring and method development. As awareness of TA toxicity increases, especially in vulnerable populations, future studies and regulatory efforts should aim to close this gap and ensure comprehensive safety evaluations.

The occurrence of PAs and TAs in honey and related apicultural products has been extensively investigated, revealing a highly complex and dynamic contamination pattern influenced by botanical origin, environmental factors, seasonal variation, and processing conditions. Honey, pollen, royal jelly, and propolis can all accumulate these naturally occurring toxins, with concentrations ranging from trace levels to several milligrams per kilogram, depending on the floral source and geographic location [55,77,78,79,80,81,82,83,84,85,86,87,88,89,90,91,92,93,94,95,96,97,98,99,100,101].

Among these, PAs are the most frequently detected, generally occurring as *N*-oxide derivatives (PANOs), which exhibit a high degree of instability during storage. Kaltner et al. (2018) demonstrated that PANOs in fortified honey decreased by 34% after only one day and by 99% after 182 days, while the corresponding parent PAs remained stable [76]. This degradation behavior highlights the necessity of timely sample analysis and proper storage to avoid underestimation of total PA content and ensure accurate risk assessments. Concentration variability is primarily determined by the botanical composition of nectar and pollen sources. Mulder et al. (2018) found PA levels in pollen ranging from 330 to 1911 µg/kg (mean = 576 µg/kg), whereas propolis and royal jelly contained only trace amounts, confirming pollen as the main vector of contamination in bee-derived products [55]. Raw honey from Germany reached up to 3313 µg/kg, with strong seasonal fluctuations and high variability even among hives from the same area [81]. Similarly, honeys from China, South Korea, Brazil, and various European countries exhibited broad ranges of PA concentrations, dominated by lycopsamine- and echimidine-type compounds, which are characteristic of Asteraceae and Boraginaceae species such as *Senecio*, *Echium*, and *Crotalaria* [82,87,93,101]. Multifloral honeys generally display a broader alkaloid spectrum, while certain monofloral honeys (e.g., thyme, thistle, *Ferula*, and *Sulla*) tend to contain moderate mean concentrations (10–25 µg/kg), occasionally exceeding these levels depending on floral purity [101]. Bee-collected pollen and bee bread frequently show substantially higher concentrations, with extreme cases in Brazilian samples reaching up to 263,849 µg/kg [98], underlining the relevance of these matrices for dietary exposure estimation.

As summarized in Table 3, a wide variety of analytical methodologies have been developed to detect and quantify PAs and TAs in honey and pollen. The majority of studies employed LLE using dilute sulfuric acid (0.05 M H_2_SO_4_), often followed by SPE cleanup with C18, SCX, or MCX sorbents to reduce matrix interferences [47,55,76,79,80,81,91,97]. Alternative techniques include QuEChERS and its variants, such as QuPPe (Quick Polar Pesticides, designed for highly polar and water-soluble pesticides) and dispersive liquid–liquid microextraction (DLLME) [77,78,88,89], as well as newer eco-efficient approaches like µSPEed [95] or the use of nanostructured sorbents based on halloysite nanotubes [92,93], which have achieved recoveries exceeding 90%. A few works also incorporated zinc reduction steps to convert PANOs into their tertiary amine forms prior to analysis [77,90,91]. Chromatographic separation is consistently carried out using reversed-phase C18 columns, occasionally C8 or polar-modified stationary phases, typically maintained between 30 and 50 °C. Detection relies primarily on LC–MS/MS platforms operated in positive electrospray ionization (ESI+) and MRM modes, offering high selectivity and sensitivity at µg/kg levels [77,78,79,80,81,82,83,84,85,86,87,88,89,90,91,92,93,94,95,96,97,98,99,100,101]. Recent studies have increasingly adopted HRMS and ion-mobility spectrometry (IMS) for non-targeted screening, enabling the detection of both regulated and emerging alkaloids [85,88,89,101]. However, as shown in Table 3, extraction efficiency, clean-up strategy, and matrix effects vary considerably between methods, and these factors may significantly influence reported concentrations, highlighting the need for inter-laboratory harmonization.

Although the presence of PAs in honey is widespread, most commercial products remain below current regulatory limits for adult consumption. Nonetheless, occasional exceedances, particularly in unblended, raw, or pollen-rich honeys, pose a potential risk to children and other sensitive groups [87,90,92]. TAs, including atropine, scopolamine, and hyoscyamine, occur less frequently but have been detected at concentrations up to several tens of µg/kg in some multifloral honeys [84,85,96,97]. Their co-occurrence with PAs in several studies supports the need for simultaneous monitoring and cumulative risk assessment approaches rather than evaluating each compound class in isolation. Overall, the data compiled in Table 3 underscore that both floral origin and environmental conditions surrounding the hives are key determinants of PA and TA contamination. The dominance of *Senecio*, *Echium*, *Crotalaria*, and *Borago* species in foraging areas consistently correlates with elevated PA content, while variations in bloom periods, pollen composition, and bee foraging patterns account for the pronounced heterogeneity observed even within single apiaries [87,92,100].

In conclusion, honey and related apicultural products constitute complex natural matrices where PAs and, to a lesser extent, TAs may accumulate through plant–pollinator interactions. While concentrations in most honeys are generally low, certain products, especially those with high pollen content or derived from PA-producing floras, can exceed safety thresholds. Analytical methodology plays a pivotal role in both reliable quantification and risk evaluation. Continued monitoring, the integration of both PA and TA determinations into standard quality control protocols, and further research on alkaloid stability, bioaccessibility, and mitigation strategies are essential to ensure consumer protection and maintain confidence in apicultural products [47,55,77,78,79,80,81,82,83,84,85,86,87,88,89,90,91,92,93,94,95,96,97,98,99,100,101].

## 4. Conclusions

As evidenced by current research, edible flowers and their derived products represent a growing category of novel foods, driven by increasing interest in natural, functional, and sustainable ingredients. While these products can provide nutritional and organoleptic benefits, they may also be exposed to a variety of contaminants, including pesticide residues, heavy metals, insects, and pathogenic microorganisms. In addition, the presence of naturally occurring toxic secondary metabolites, such as TAs and PAs, has raised concerns regarding consumer safety. Several studies have confirmed that fresh flowers may contain these toxins, with borage flowers being particularly noteworthy due to its known capacity to produce PAs. Consequently, its inclusion in legislation regulating maximum allowable levels of these substances should be considered. Other floral species have also shown contamination, possibly resulting from cross-contamination during cultivation, harvesting, or processing. Regarding flower-based derived products, such as infusions and food supplements, a significant number of studies have focused on the detection of these alkaloids, given their tendency to appear in such matrices. The wide range of concentrations reported may be attributed to the intentional inclusion of alkaloid-producing plants in formulations, cross-contamination with such species, or horizontal transfer mechanisms. Finally, in the case of honey, high concentrations of PAs have been found in numerous samples. TAs, however, have received less attention in both food supplements and apicultural products, despite positive findings in some cases. This highlights the need to broaden the analytical scope to include both groups of alkaloids in future research.

Furthermore, flower-derived products have been the focus of a greater number of analytical studies compared to fresh flowers, resulting in the development of a wider range of validated methodologies. These include miniaturized techniques and the application of novel sorbents, reflecting a higher level of analytical sophistication. In contrast, research specifically addressing fresh flowers remains limited, underscoring a methodological gap that merits further investigation. Overall, the findings underscore the importance of continued research to ensure the safety of these emerging products, particularly in light of their increasing consumption and market presence.

## Figures and Tables

**Table 1:** Alerts notified by the RASFF in edible flowers [14].

Subject	Notification Type and Date	Notified by	Countries Concerned	Risk and Action Taken
**Pesticides**				
Ethylene oxide in organic chamomile flowers from Egypt	Alert 20 December 2024	Germany	Austria, Bulgaria, Canada, Czech Republic Egypt, Finland, Germany, INFOSAN, Italy, Poland, Slovakia, Spain, Sri Lanka, Switzerland, United Kingdom	Potentially serious Withdrawal from the market, informing authorities, informing recipient(s) and detained by operator.
Unauthorised substance prometryn in organic whole silver linden flowers (*Tilia argentea*) from Bulgaria	Alert 3 September 2024	Austria	Austria, Bulgaria, Germany, INFOSAN, Italy, Japan, Romania, Switzerland	Serious Informing recipient(s), withdrawal from recipient(s), destruction and monitoring of the withdrawal
Unauthorized substance chlorpyrifos ethil in hibiscus flower from Nigeria	Information for attention 23 March 2023	Italy	Italy, Nigeria, Serbia	Potentially serious Informing authorities and withdrawal from recipient(s)
Unauthorized substance chlorpyrifos in organic daisy flower from Albania	Information notification follow-up 10 March 2023	France	Albania, France, Germany, Spain	Potentially serious Withdrawal from the market and recall from costumers
Unauthorized substance chlorpyrifos and thiophanate methyl in chamomile flower	Information for attention 9 March 2023	France	Egypt, France, Germany, INFOSAN	Potentially serious Recall from consumer and withdrawal from recipient(s)
**Pathogens**				
*Salmonella* spp. in organic red clover flowers from Albania, via Croatia	Alert 30 May 2025	Germany	Albania, Croatia, Germany, INFOSAN	Informing authorities, physical treatment and detained by operator
*Salmonella* spp. in lavender flowers from France	Alert 2 May 2025	Germany	France, Germany	Informing authorities and detained by operator
*Salmonella* Typhimurium in organic linden flower infusion from Bulgaria, via Austria	Information notification for follow-up 17 February 2021	Finland	Austria, Bulgaria, Finland, Germany, Italy, Poland, Switzerland	Not serious Withdrawal from the market
**Alkaloids**				
Chamomile flowers above the maximum level of pyrrolizidine alkaloids	Alert 12 December 2025	Switzerland	Egypt, Germany, INFOSAN, Switzerland	Recall from consumer

**Table 2:** Analytical procedures for the determination of pyrrolizidine and tropane alkaloids and their concentration levels found in edible flowers and edible flower-based products (2018–2025).

Sample Type	N° of PAs/TAs	Sample Preparation	Analysis	LOD/LOQ	Recoveries (%)	Range of PAs/TAs Found	Ref.
**Fresh edible flowers**							
Borage (aerial parts)	9 PAs	SLE with MeOH 70%	UHPLC-Q-TOF/MS ESI positive Column: C18 (40 °C)	0.2–2/0.6–6 ng/mL	-	-	[38]
*Chrysanthemum morifolium* flowers	1 PA	HPE with acetic acid	HPLC-TQ-MS/MS ESI positive (MRM mode) Column: C18	-	-	-	[39]
Borage (flowers and aerial parts)	18 PAs	SLE with H_2_SO_4_ (0.05 M) two times and adjusted the pH to 6–7 followed by SPE-C18	UHPLC-QTrap-MS/MS ESI positive (MRM mode) Column: C18 (25 °C)	-/25–50 μg/kg	85–112%	-	[40]
Borage flowers	17 PAs	SLE with 0.2% FA and 10% MeOH in H_2_O	UHPLC-QTrap-MS/MS ESI positive (MRM mode) Column: C18 (25 °C)	-/25–50 μg/kg	13–118%	77–88,602 µg/kg	[41]
**Dry edible flower infusions**							
Chamomile	27 PAs	QuEChERS (clean-up with PSA and ENVI-Carb sorbents)	UHPLC-TQ-MS/MS ESI positive (MRM mode) Column: C18 (40 °C)	0.03–0.48/0.10–1.61 μg/kg	88–107%	2.6–212 μg/kg	[42]
Different flower species	4 TAs	SLE with ACN:H_2_O (3:2 *v*/*v*) with 0.2% FA	UHPLC-TQ-MS/MS ESI positive (MRM mode) Column: C18 (40 °C)	<25/<25 μg/kg	83–105%	25–69 μg/kg	[43]
Chamomile	44 PAs	SLE with H_2_SO_4_ (0.05 M) followed by SPE-SCX	HPLC-TQ-MS/MS ESI positive (MRM mode) Column: C18 (30 °C)	0.1–7.0/0.1–27.9 µg/kg	52–152%	4.1–13.2 μg/kg	[44]
Lavender, chamomile, chrysanthemum and hibiscus	21 PAs	SLE with 50% MeOH solution with 0.05 M H_2_SO_4_ followed by SPE-MCX	UHPLC-TQ-MS/MS ESI positive (MRM mode) Column: C18 (40 °C)	0.1–3/0.3–9 µg/kg	86–101%	0.002–0.22 mg/kg	[45]
Chrysanthemum	14 PAs	SLE with H_2_SO_4_ (0.1 M) followed by SPE-MCX.	UHPLC-TQ-MS/MS ESI positive (MRM mode) Column: HSS T3 (40 °C)	0.001–0.4/1–5 µg/kg	68–110%	<LOQ-5.2 µg/kg	[46]
Rose hip and chamomile	17 PAs	SLE with 2% FA and adjusted the pH to 10 with 25% ammonia solution followed by automated SPE.	UHPLC-TQ-MS/MS ESI positive (MRM mode) Column: BEH C18 (40 °C)	>2.0/>5.0 µg/kg	62–147%	28–221 µg/kg	[47]
Chamomile	28 PAs and 2 TAs	QuEChERS (clean-up with MgSO_4_, PSA, C18 and GCB sorbents)	LC-Q-Orbitrap-HRMS H-ESI positive (MRM mode) Column: C18 (30 °C)	PAs: -/5 µg/kg TAs: -/5 µg/kg	PAs: 87–111% TAs: 90–111%	-	[48]
Scented teas (mixture of tea with flowers)	10 PAs	SLE with 10% ammonium hydroxide, addition of MeOH:dichloromethane (1:1), evaporation and reconstitution in MeOH: H_2_O (1:1), pH adjusted to neutral with FA, followed by SPME-MIP	UHPLC-Q-TOF-MS ESI positive (MRM mode) Column: HSS T3 (35 °C)	0.08–0.54/0.26–1.77 µg/L	91–108%	10.8–139.6 μg/kg	[49]
Chamomile	14 PAs	SLE with H_2_SO_4_ (0.05 M) followed by SPE-MCX	UHPLC-TQ-MS/MS SIM mode Column: XB-C18 (25 °C)	-	-	-	[50]
Chamomile and *Echinacea angustifolia*	4 TAs	QuEChERS (clean-up with PSA and MgSO_4_)	UHPLC-TOF-MS ESI positive Column: C18 (20 °C)	2.5–10/5–15 µg/kg	82–104%	-	[51]
Chamomile	35 PAs	SLE with H_2_SO_4_ (0.05 M) followed by SPE-MCX	UHPLC-TQ-MS/MS ESI positive (MRM mode) Column: C8	0.5/1–5 µg/kg	70–106%	5–268 µg/kg	[52]
Chamomile	35 PAs	SLE with H_2_SO_4_ (0.05 M) in H_2_O:MeOH (1:1 *v*/*v*) followed by SPE-MCX	UHPLC-TQ-MS/MS ESI positive (MRM mode) Column: C8 (40 °C)	0.1–1.0/0.6–3.0 µg/kg	87–103%	7.7 µg/kg	[53]
Chamomile	36 PAs	SLE with H_2_O:MeOH 0.2% FA (1:1 *v*/*v*)	UHPLC-TQ-MS/MS ESI positive (MRM mode) Column: C18 (40 °C)	<2/2–10 µg/kg	43–108%	34.6–697.2 µg/kg	[54]
**Liquid edible flower infusions**							
Chamomile	38 PAs	Infusion with boiling water followed by SPE-C18	UHPLC-TQ-MS/MS ESI positive (MRM mode) Column: C18 (50 °C)	0.2–3.8/- μg/kg	30–122%	LOQ-18.79 μg/L	[55]
Borage, coltsfoot, comfrey, climbing groundsel, sun hemp	70 PAs	Infusion with boiling water	UHPLC-TQ-MS/MS ESI positive (MRM mode) Column: C18 (50 °C)	0.01–0.02/0.05 μg/L	73–107%	30.7–1120 μg/L	[56]
Edible flower	21 PAs	Infusion with boiling water followed by µSPEed-C18 purification	UHPLC-IT-MS/MS ESI positive (MRM mode) Column: C18 (25 °C)	0.1–0.3/0.3–1.0 µg/L	79–97%	23–41 µg/L	[57]
Chamomile	2 TAs	Infusion with boiling water followed by µSPEed-PS/DVB purification	HPLC-TQ-MS/MS ESI positive (MRM mode) Column: C18 (30 °C)	0.02–0.05/0.06–0.15 ng/mL	94–106%	0.08–0.18 ng/mL	[58]
Chamomile and different flower species	20 PAs and 2 TAs	Infusion with boiling water followed by SPE-C18	UHPLC-TQ-MS/MS ESI positive (MRM mode) Column: C18 (40 °C)	PAs: 0.04–0.08/0.07–0.14 µg/L TAs: 0.003–0.004/0.005–0.007 µg/L	PAs: 88.3–116.0% TAs: 93.6–114.0%	PAs: LOD–2.82 µg/L TAs: LOD–2.4 µg/L	[59]
Chamomile	21 PAs	Infusion with boiling water followed by µSPEed-C18 purification	UHPLC-IT-MS/MS ESI positive (MRM mode) Column: C18 (25 °C)	-/-	76–101%	-	[60]
*Echinacea purpurea*	2 TAs	Infusion with boiling water followed by µSPEed-PS/DVB purification	HPLC-TQ-MS/MS ESI positive (MRM mode) Column: C18 (30 °C)	0.02–0.05/0.06–0.18 ng/mL	73–114%	-	[61]
Different flower species	118 PAs	Infusion with boiling water followed by SALLE	UHPLC-Q-Orbitrap-HRMS/MS ESI positive (HRMS mode) Column: Polar C18 (40 °C)	LOD: 0.6–30 µg/kg	69–113%	0.87–127.15 µg/kg	[62]
Edible flower	21 PAs	Infusion with boiling water followed by µSPEed-C18 purification	UHPLC-IT-MS/MS ESI positive (MRM mode) Column: C18 (30 °C)	0.03–0.07/0.09–0.2 µg/L	87–97%	0.16–0.2 µg/L	[63]
Herb infusion containing hibiscus	62 PAs and 2 TAs	Infusion with boiling water followed by SPE-Strata X	UHPLC-TQ-MS/MS ESI positive (MRM mode) Column: C18 (50 °C)	PAs: 0.01–0.05/0.05 ng/mL TAs: 0.01–0.05/0.05 ng/mL	PAs: 73–126 % TAs: 73–126 %	-	[64]
**Food supplements**							
Herbal supplements containing flowers	38 PAs	SLE with H_2_SO_4_ (0.05 M) followed by SPE-C18	UHPLC-TQ-MS/MS ESI positive (MRM mode) Column: C18 (50 °C)	0.3–2.3/- μg/kg	45–98%	7.2–4674 µg/kg	[55]
Extract and tablets containing different flower species	4 TAs	SLE with ACN:H_2_O (3:2 *v*/*v*) with 0.2% FA	UHPLC-TQ-MS/MS ESI positive (MRM mode) Column: C18 (40 °C)	<25/<25 μg/kg	83–105%	-	[43]
Liqueurs, elixirs and herbal juices containing different flower species	30 PAs	LLE with H_2_SO_4_ (0.05 M) and purification by SPE-SCX.	HPLC-MS/MS ESI positive (MRM mode) Column: C18 (25 °C)	-	-	0.21–3121 μg/kg	[65]
*Gynura japonica* food supplement	6 PAs	SLE with 1% FA followed by LLE with dichloromethane.	DART-IT-MS Positive ion mode	0.55–0.85/1.83–2.82 ng/mL	89–112%	2130–234,300 μg/kg	[66]
*Chyrantemum morifolium* food supplement	28 PAs	SLE with 50% MeOH with 0.05 M H_2_SO_4_ followed by SPE-MCX	HPLC-TQ-MS/MS ESI positive (MRM mode) Column: C18 (40 °C)	0.03–2.1/0.1–6.5 µg/kg	67–151%	-	[67]
Food supplements including different flower species	118	SLE with H_2_SO_4_ (0.05 M) followed by SALLE	UHPLC-Orbitrap- MS/MS ESI positive (HRMS mode) Column: C18 (40 °C)	0.6–30/- µg/kg	69–113%	-	[62]
Food supplements including different flower species	29 PAs and 2 TAs	QuEChERS (without clean-up step)	HPLC-TQ-MS/MS ESI positive (MRM mode) Column: C18 (40 °C)	PAs: -/0.5–10 ng/g TAs: -/1 ng/g	PAs: 86–104% TAs:90–93%	PAs: 0.62–1097 μg/kg TAs: 1.5–1.8 μg/kg	[68]
Flower-based extracts	21 PAs and 2 TAs	Evaporation and reconstitution in H_2_O followed by µSPEed (synthesized SM-C18 cartridges)	UHPLC-IT-MS/MS ESI positive (MRM mode) Column: C18	PAs: 0.2–0.6/0.6–2 ng/g TAs: 0.3 ng/g-1–1.1 ng/g	PAs: 91–97% TAs:93–94%	PAs: - TAs: 14.8–20.8 μg/kg	[69]

C8: Octyl bonded silica; C18: Octadecyl bonded silica; DART: Direct analysis in real time; ESI: Electrospray ionization; FA: Formic acid; GCB: Graphitized carbon black; H-ESI: Heated-electrospray ion source; HPLC: High-performance liquid chromatography; HRMS: High resolution mass spectrometry; HSS T3: High Strength Silica reversed phase; IT: Ion-trap; LC: Liquid chromatography; LLE: Liquid–liquid extraction; LOD: Limit of detection; LOQ: Limit of quantification; MCX: Mixed-mode sorbent with non-polar and cation exchange properties; MeOH: Methanol; MIP: Molecularly imprinted polymer; MRM: Multiple reaction monitoring; MS/MS: Tandem mass spectrometry; MS: Mass spectrometry; PAs: Pyrrolizidine alkaloids; PS/DVB: Polystyrene/divinylbenzene; PSA: Primary secondary amine; Q: Single quadrupole; QTOF: Quadrupole time-of-flight; QTRAP: Hybrid triple quadrupole-linear ion trap; QuEChERS: Quick, easy, cheap, effective, rugged and safe; SALLE: Salting-out assisted liquid–liquid extraction; SCX: Strong cation Exchange; SIM: Selected ion monitoring; SLE: Solid–liquid extraction; SPE: Solid-phase extraction; SPME: Solid phase microextraction; TAs: Tropane alkaloids; TQ: Triple quadrupole; UHPLC: Ultra-high-performance liquid chromatography.

**Table 3:** Analytical procedures for the determination of pyrrolizidine and tropane alkaloids and their concentration levels found in honey and honey-based products (2018–2025).

Sample Type	N° of PAs/TAs	Sample Preparation	Analysis	LOD/LOQ	Recoveries (%)	Range of PAs Found	Ref.
**Honey and related products**							
Honey	25 PAs	LLE with H_2_SO_4_ (0.05 M) followed by SPE-SCX	HPLC-TQ-MS/MS ESI positive (MRM mode) Column: polar-reversed phase (30 °C)	0.01–1.60/0.03–5.40 µg/kg	49–121%	-	[76]
Honey and pollen	38 PAs	LLE with H_2_SO_4_ (0.05 M) followed by SPE-C18	UHPLC-TQ-MS/MS ESI positive (MRM mode) Column: C18 (50 °C)	0.2–0.6/- μg/kg	72–122%	48–1911 μg/kg	[55]
Honey	9 PAs	Dilution with acidified water (0.25 M acetic acid), addition of zinc and pH adjusted to 9.5 followed by DLLME with chloroform and isopropyl alcohol	UHPLC-QTrap-MS/MS ESI positive (MRM mode) Column: C18 (30 °C)	-/0.03–0.06 µg/kg	63–103%	0.2–17.5 μg/kg	[77]
Honey	7 PAs	QuPPe (without clean-up step)	HPLC-QTrap-MS/MS ESI positive (MRM mode) Column: C18	-/8–18 µg/kg	50–100%	43–75 μg/kg	[78]
Honey	12 PAs	LLE with H_2_SO_4_ (0.05 M) followed by SPE-MCX	HPLC-QTOF-MS/MS ESI positive (HRMS mode) Column: C18 (40 °C)	0.2–0.6/0.5–1.3 µg/kg	79–104%	1.4–14.2 μg/kg	[79]
Honey	25 PAs	LLE with H_2_SO_4_ (0.05 M) followed by SPE-SCX	HPLC-TQ-MS/MS ESI positive (MRM mode) Column: reversed phase (phenyl)-polar (40 °C)	0.01–0.19/0.03–0.59 µg/kg	82–121%	2.4–446 μg/kg	[80]
Honey	17 PAs	LLE with 0.05 M H_2_SO_4_: MeOH (85:15, *v*/*v*) followed by SPE-MCX	HPLC-TQ-MS/MS ESI positive (MRM mode) Column: C18	-	-	0.2–281.1 μg/kg	[81]
Honey	2 PAs	LLE with H_2_SO_4_ (0.05 M) followed by SPE-SCX	HPLC-DAD λ: 223 nm Column: C18	-	-	-	[82]
Honey	11 TAs	SLE with MeOH/H_2_O/FA 75/25/0.4 and clean-up with MgSO_4_ and GBC	HPLC-HRMS HESI positive and negative (Full scan) Column: ACE HILIC-A (25 °C)	-/20–40 µg/kg	71–120%	27 µg/kg	[83]
Honey	2 TAs	SALLE	UHPLC-TQ-MS/MS ESI positive (MRM mode) Column: HILIC core–shell (40 °C)	0.002–0.003/0.01 µg/kg	87–106%	0.012 µg/kg	[84]
Honey	26 PAs	LLE with 6.5 mmol/L NH_4_OH	UHPLC-IM- QTOF-MS/MS ESI positive (HRMS mode) Column: C18 (50 °C)	1–7/10–20 µg/kg	75–120%	0–141.8 μg/kg	[85]
Honey and pollen	8 PAs	LLE with 70% MeOH in H_2_O acidified with 2% FA	HPLC-QTrap-MS/MS ESI positive (MRM mode) Column: C18 (30 °C)	-	-	0–623 µg/kg (honey) 24.9–221 mg/kg (pollen)	[86]
Pollen	44 PAs	SLE with H_2_SO_4_ (0.05 M) followed by a second extraction followed by SPE-C18	UHPLC-MS/MS ESI positive (dMRM mode) Column: C18 (50 °C)	0.09–3.6/0.26–7.9 µg/kg	63–120%	0.48–48,400 ng/g	[87]
Honey	7 PAs	QuEChERS (clean-up with PSA)	UHPLC-IM- QTOF-MS/MS ESI positive (HDMS^E^ mode) Column: C18 (50 °C)	-/1–20 µg/kg	61–120%	-	[88]
Honey	30 PAs	QuEChERS (without clean-up step)	Nano-LC-Orbitrap-MS/MS ESI positive (HRMS mode) Capillary column: C18 (50 °C)	-/0.027–11 µg/kg	-	0.14–74 μg/kg	[89]
Honey	17 PAs	SLE with FA in water (2% *v*/*v*) and adjusted the pH to 10 with 25% ammonia solution followed by automated SPE	UHPLC-TQ-MS/MS ESI positive (MRM mode) Column: C18 (40 °C)	>0.5 µg/kg/>1.5 µg/kg	62–147%	1.2–103 µg/kg	[47]
Honey	17 PAs and 2 TAs	SLE with H_2_SO_4_ (0.05 M) and zinc powder followed by SPE-MCX	HPLC-Q-MS ESI positive (SIM mode) Column: C18 (30 °C)	PAs: 0.05–0.17/0.17–0.58 µg/kg TAs: 0.11–0.15/0.36–0.49 µg/kg	PAs: 81–106% TAs: 89–102%	PAs: 2.2–147 µg/kg TAs: -	[90]
Honey and pollen	30 PAs	SLE with H_2_SO_4_ (0.05 M) in MeOH followed by SPE-MCX	LC-MS/MS ESI positive (MRM mode) Column C18 (40 °C)	-	-	0.9–72.6 µg/kg	[91]
Pollen	20 PAs and 2 TAs	SLE with H_2_SO_4_ (0.1 M) and zinc powder followed by QuEChERS (clean-up with PSA and MgSO_4_)	UHPLC-TQ-MS/MS ESI positive (MRM mode) Column: C18 (40 °C)	PAs: 0.04–0.08/0.07–0.14 µg/L TAs: 0.003–0.004/0.005–0.007 µg/L	PAs: 73.1–106.4% TAs: 78.0–91.2%	PAs: LOD-271 µg/kg TAs: LOD–10.9 µg/kg	[59]
Honey	4 PAs	Dilution with water followed by SPE-HNT-SO_3_H	UHPLC-TQ-MS/MS ESI positive (MRM mode) Column: C18 (40 °C)	3.2–4.8/6.7–12.3 µg/L	55–92%	-	[92]
Honey	4 PAs	LLE with FA (0.05 M) followed by SPE with alkyl-sulfonated halloysite nanotube sorbents (HNT-PhSO_3_H or HNT-MPTMS-SO_3_H)	UHPLC-TQ-MS/MS ESI positive (MRM mode) Column: C18 (50 °C)	0.6–1.2/1.9–3.6 µg/L	78–101%	-	[93]
Honey	32 PAs	SLE with H_2_SO_4_ (0.05 M) followed by SPE-MCX	UHPLC-MS/MS ESI positive (MRM mode) Column: C18 (30 °C)	0.06–0.25/0.22–0.82 µg/kg	66–91%	2.2–207.0 µg/kg	[94]
Honey and pollen	35 PAs	*Honey*: SLE with H_2_SO_4_ (0.1 M) followed by QuEChERS (without clean-up step) *Pollen*: SLE with H_2_SO_4_ (0.05 M) followed by SPE-MCX	UHPLC-TQ-MS/MS ESI positive (MRM mode) Column: C8	0.5/1–5 µg/kg	70–106%	1–121.1 µg/kg (honey) 6–10,168 µg/kg (pollen)	[52]
Honey and pollen	118 PAs	*Honey*: dilution with water followed by SALLE *Pollen*: SLE with H_2_SO_4_ (0.05 M) followed by SALLE	UHPLC-Q-Orbitrap- MS/MS ESI positive (HRMS mode) Column: Polar C18 (40 °C)	LOD: 0.6–30 µg/kg	69–113%	0–37.3 μg/kg	[62]
Honey	21 PAs and 2 TAs	SLE with H_2_SO_4_ (0.05 M) followed by µSPEed-PS/DVB	UHPLC-IT-MS/MS ESI positive (MRM mode) Column: C18 (30 °C)	PAs: 0.12–0.3/0.4–1.0 µg/kg TAs:	PAs: 72–100% TAs: 81–97%	PAs: 24–159 µg/kg TAs: 3.7–18.6 µg/kg	[95]
Honey	2 TAs	SPE (methacrylic acid synthesized polymer)	HPLC-TQ-MS/MS ESI positive (MRM mode) Column: C18 (30 °C)	0.19–0.56/0.625–1.875 ng/g	71–95%	1.4–7.2 μg/kg	[96]
Honey	30 PAs	SLE with H_2_SO_4_ (0.05 M) followed by SPE-SCX	UHPLC-MS/MS ESI positive (MRM mode) Column C18 (50 °C)	-	-	0.6–85 μg/kg	[97]
Brazilian bee bread and pollen	8 PAs	SLE with MeOH:H_2_O (70:30 *v*/*v*) acidified with 2% of FA followed by a dilution 1:1 with mobile phase	LC-MS/MS ESI positive (MRM mode) Column: C18 (30 °C)	0.1–1.0/0.2–1.5 µg/kg	-	268–263,849 μg/kg	[98]
Honey and pollen	28 PAs	*Honey*: SLE with EDTA buffer (oH 2.4) followed by online-SPE *Pollen*: SLE with EDTA buffer (oH 2.4) and acetonitrile followed by LLE with hexane. Finally, the extracts were purified with online-SPE	UHPLC-TQ-MS/MS ESI positive (MRM mode) Column: C8 (45 °C)	0.5–0.5/0.25–1.0 µg/kg	82–117%	*Honey*: 0.28–117.38 μg/kg *Pollen* LOQ-14,534 μg/kg	[99]
Honey	35 PAs	QuEChERS (without clean-up step)	UHPLC-TQ-MS/MS ESI positive (MRM mode) Column: C8	-/1.0 µg/kg	70–120%	1–50 µg/kg	[100]
Honey	35 PAs	SLE with H_2_SO_4_ (0.05 M) in H_2_O:MeOH (1:1 *v*/*v*) followed by SPE-MCX	UHPLC-TQ-MS/MS ESI positive (MRM mode) Column: C8 (40 °C)	0.6–3.0/0.6–3.0 µg/kg	50–77%	2.7 µg/kg	[53]
Honey	24 PAs	SLE with 2% FA followed by SPE-MCX	UHPLC-QTrap-MS/MS ESI positive (MRM mode) Column: HSS T3 (40 °C)	0.015–0.3/0.05–1.00 µg/kg	65–103%	3.2–20.5 µg/kg	[101]

C8: Octyl bonded silica; C18: Octadecyl bonded silica; DAD: Diode array detection; DLLME: Dispersive liquid–liquid microextraction; dMRM: Dynamic multiple reaction monitoring mode; ESI: Electrospray ionization; FA: Formic acid; HNT-SO_3_H: Sulfonated halloysite nanotubes; HPLC: High-performance liquid chromatography; HRMS: High resolution mass spectrometry: HSS T3: High Strength Silica reversed phase; IM: Ion mobility; IT: Ion-trap; LC: Liquid chromatography; LLE: Liquid–liquid extraction; LOD: Limit of detection; LOQ: Limit of quantification; MCX: Mixed-mode sorbent with non-polar and cation exchange properties; MeOH: Methanol; MRM: Multiple reaction monitoring; MS/MS: Tandem mass spectrometry; MS: Mass spectrometry; PAs: Pyrrolizidine alkaloids; PS/DVB: Polystyrene/divinylbenzene; PSA: Primary secondary amine; Q: Single quadrupole; QTOF: Quadrupole time-of-flight; QTrap: Hybrid triple quadrupole-linear ion trap; QuEChERS: Quick, easy, cheap, effective, rugged and safe; QuPPe: Quick Polar Pesticides; SALLE: Salting-out assisted liquid–liquid extraction; SCX: Strong cation Exchange; SIM: Selected ion monitoring; SLE: Solid–liquid extraction; SPE: Solid-phase extraction; TQ: Triple quadrupole; UHPLC: Ultra-high-performance liquid chromatography.

## Data Availability

No new data were created or analyzed in this study.
